# Contingent association between the size of the social support network and osteoporosis among Korean elderly women

**DOI:** 10.1371/journal.pone.0180017

**Published:** 2017-07-12

**Authors:** Seungwon Lee, Da Hea Seo, Kyoung Min Kim, Eun Young Lee, Hyeon Chang Kim, Chang Oh Kim, Yoosik Youm, Yumie Rhee

**Affiliations:** 1 Department of Sociology, Yonsei University, Seoul, South Korea; 2 Department of Endocrinology and Metabolism, Inha University School of Medicine, Incheon, South Korea; 3 Department of Internal Medicine, Seoul National University Bundang Hospital, Seongnam, South Korea; 4 Division of Endocrinology and Metabolism, Department of Internal Medicine, Seoul St. Mary's Hospital, The Catholic University of Korea College of Medicine, Seoul, South Korea; 5 Department of Internal Medicine, Yonsei University College of Medicine, Seoul, South Korea; Nanjing Medical University, CHINA

## Abstract

**Objective:**

To investigate the association between the number of personal ties (or the size of the social support network) and the incidence of osteoporosis among older women in Korea.

**Methods:**

Data from the Korean Urban Rural Elderly Study were used. Bone density was measured by dual-energy X-ray absorptiometry at the lumbar spine (L1–L4) and femur neck. T-score, the standardized bone density compared with what is normally expected in a healthy young adult, was measured and the presence of osteoporosis was determined, if the T-score was < -2.5. The social support network size was measured by self-responses (number of confidants and spouse).

**Results:**

Of the 1,846 participants, 44.9% were diagnosed with osteoporosis. The association between the social support network size and the incidence of osteoporosis was curvilinear in both bivariate and multivariate analyses. Having more people in one’s social support network size was associated with lower risk of osteoporosis until it reached around four. Increasing the social support network size beyond four, in contrast, was associated with a higher risk of osteoporosis. This association was contingent on the average intimacy level of the social network. At the highest average intimacy level (“extremely close”), increasing the number of social support network members from one to six was associated with linear decrease in the predicted probability of osteoporosis from 45% to 30%. However, at the lowest average intimacy level (“not very close”), the predicted probability of osteoporosis dramatically increased from 48% to 80% as the size of the social network increased from one to six.

**Conclusion:**

Our results show that maintaining a large and intimate social support network is associated with a lower risk of osteoporosis among elderly Korean women, while a large but less-intimate social relationship is associated with a higher risk.

## Introduction

Osteoporosis has no distinctive symptoms until it causes fractures, and once progressed, it is difficult to recover the optimal bone density [1]. Therefore, it is important to recognize the risk factors and to take preventive action before it is too late. The finding of a systematic association between maintaining a certain type of social relationship and having osteoporosis could help adopt initiatives to reduce the prevalence of osteoporosis.

Social support, which can be defined as the various types of resources provided by other people, has been widely used in health studies to explain diverse health statuses [2]. Social network analysis extended this prior approach by focusing on the structures of social ties (networks). A social network refers to the web of social relationships around people, which has diverse functions, such as providing social support, access to material sources, and opportunities for social engagement and participation and developing social norms [3]. Among the various structural characteristics of social networks, health research has most often focused on the size of social networks. A study of 6,928 American adults showed that the age-adjusted risk ratio of mortality for female participants with large network was 2.8 times lower than that of socially isolated individuals [4]. Similar results demonstrating the preventive effect of social support networks on mortality have been replicated by several prospective and experimental studies [5].

Although the size of the social support network as a risk factor on bone density has rarely been studied, some studies have suggested that being married could be beneficial to bone health. Korean older women who lived with their spouses approximately had 20% point lower prevalence of osteoporosis than those who did not [6]. In addition, the odds of having a hip fracture was 1.4 times higher for single Swedish women aged over 50 years than those for married or cohabiting women [7]. Similar results were observed in a study of middle-aged Norwegian women [8]. Nabipour et al. showed that Australian elderly men who are married had a higher bone mineral content than those who lived alone [9]. Further, a recent study on Chinese elderly individuals assessed the relationship between osteoporosis and the amount of received social support [10]. In this study, three dimensions of social support (objective support, subjective support, and utilization of support) were measured by asking 10 questions. The results showed that osteoporosis patients had a lower level of social support in every dimension.

Supportive networks provide older women with material, emotional, or informational support to maintain a healthy lifestyle and receive proper care. Social participation also leads them to be physically more active, eat more, and be exposed to sunlight, which can prevent bone loss in older women. In addition, while stressful life events might accelerate bone loss [11], social care from network members can mitigate these detrimental effects. However, inverse association between network size and bone loss may be observed, if the network is too large. While social support networks are a source of various social resources, they can also represent a social burden that requires additional responsibilities and costs to maintain, particularly if the social ties are not perceived as being”close” [12]. Having too many not-so-close network members can potentially increase stress in older women.

When social networks consist of not-so-close friendships, they may even be considered stressful or irritating [13–15]. These negative effects are usually caused by envy, invasion of privacy, embarrassment, interpersonal conflicts, and a sense of dependence [16–19]. Even well-intended social support can be stressful if the support is not reciprocal; in other words, when people are unable to return benefits received from others [20–22]. This issue is particularly noticeable for women, because women tend to be more sensitive to the content of social relationships than men [23,24]. Closeness with network members and duration of relationships have a stronger influence on the perceived adequacy of social support for women than for men [25]. Further, research suggests that marital quality rather than marriage itself was significant for women, while merely being married had a significantly positive effect on bone density for men [26]. In short, it is plausible that the association between social support network size and osteoporosis is contingent on how close people feel about their network members, particularly among older women.

Based on the above findings, we proposed the following two hypotheses regarding the association between the size of social support networks and the incidence of osteoporosis without assuming any causal direction. First, we expected that the association between the size of social support networks and the incidence of osteoporosis would be U-shaped: larger social support networks would be related to lower odds of having osteoporosis until a certain number of social network member was reached; after the ideal number of the social support network members beyond which negative association changed into positive association, a larger network size would be associated with a higher risk of having osteoporosis. Second, the U-shaped relationship would be linear after controlling for the closeness of social network relationships. Large and close social support networks were expected to be related to a lower probability of having osteoporosis in a linear pattern, while large and non-close social ties would be associated with higher odds of having osteoporosis because of the burdens of a large social network.

## Method

### Data

To discover the relationship between the social support network and osteoporosis, data from the Korean Urban Rural Elderly study (KURE) were used. KURE is an ongoing community-based cohort study on health, aging, and common geriatric disorders of Korean elderly individuals aged over 65 years, which began in 2012 [27]. While study participants will be followed up after four years, the baseline data collected from 2012 to 2014 were used in the present study.

### Recruitment

Subjects were recruited from three urban administrative districts of Seoul and one rural district of Incheon in Korea. Recruitment was conducted using the following methods: recruiters visited random streets and houses, participants visited local government health facilities, participants contacted after seeing promotional posters for the study, and acquaintances of study participants were recruited.

All subjects were capable of communication with research teams allowing face-to-face survey interview. Demographic, physical/clinical, nutrition, lifestyle, psychological, daily activity information, as well as diverse types of social support (i.e. emotional, instrumental, financial and informational support), and social network characteristics were collected from the survey. Face-to-face survey interviews were conducted by professional interviewers in survey agency. They had been trained for at least two days to follow the standardized protocol of the study, so that the survey results do not vary by interviewer. The training included both lecture about the study protocol and rehearsing for the actual interview. The training was conducted by the qualified researchers participating in the KURE study.

### Instrumentation

Laboratory and biochemical tests, including bone mineral density, were conducted. Bone density was measured by the dual-energy X-ray absorptiometry (Hologic QDR 4500A, Waltham, USA), at two locations, the lumbar spine (L1–L4) and femur neck. The lowest T-score of these two parts was selected to diagnose osteoporosis. The presence of osteoporosis was determined using WHO guidelines, which recommend that those with a T-score under -2.5 be diagnosed with osteoporosis [28].

The questionnaire module used to identify social support networks in KURE was identical to that of the Korean Social Life, Health and Aging Project (KSHAP) and has been validated in several studies on the older Korean population [29,30]. KSHAP is a population-based longitudinal study in Korea that examines health determinants, including the social network in the elderly. It was designed to be comparable to the National Social Life, Health and Aging Project (NSHAP) in the U.S. [31]. Following the General Social Survey, KURE used the following question to identify social support members because of the clearness and validity [32,33]. “From time to time, most people discuss things that are important to them with others. For example, these may include good or bad things that happen to you, problems you are having, or important concerns you may have. Looking back over the last 12 months, who are the people with whom you most often discussed things that were important to you?” Respondents could mention up to five confidants with whom they discussed important matters. In addition to these possible five discussion partners, spouse could be added as an additional network member if the respondent was married. Thus, the social support network size ranged from zero to six.

Subjective intimacy with network members was measured by asking the following question about each member of the subject’s social support network: “How close do you consider your relationship with (the network member)?” Respondents chose from four possible answers, “not very close,” “somewhat close,” “very close,” and “extremely close.” Each answer was coded as an integer, so that 0 is for “not very close”, 1 is for “somewhat close”, 2 is for “very close”, and 3 is for “extremely close”. The average intimacy level of a participant’s social support network was measured by calculating the average of these values. Thus, the average intimacy level varied from zero to three.

Other than the social support network size and average of subjective intimacy, possible covariates predicting osteoporosis were controlled for in regression models. Because marital status was associated with bone density and fracture in previous studies [6–9], we also controlled for marital status. This variable was categorized into two groups: (1) those who were currently not married (widowed, divorced or never married) and (2) those who were currently married. Age and anthropometric variables such as body mass index (BMI) were included in the model. BMI was calculated as weight divided by the square of height in meters. Height was measured to the nearest 0.1cm using a stadiometer (DS-102, JENIX, Seoul, South Korea) at an upright position, and weight was measured to the nearest 0.1kg using an electronic scale (DB-150, CAS, Seoul, South Korea). Retrospective measures were based on self-response. Socio-economic status related variables were also controlled for in the model, including living area (urban / rural), annual income (less than 10,000 dollars / 10,000 dollars to 20,000 dollars / more than 20,000 dollars), and educational level (no schooling / elementary school / middle school or higher). Additionally, health-related behaviors controlled for included the frequency of exercise (no exercise / one to four times a week / more than five times a week), smoking (non-smoker / ex-smoker / current smoker), and drinking alcohol (no drinking / less than once a week / more than once a week).

We also believe that there exists a reverse causation between the size of social support network and the incidence of osteoporosis: while social support network is expected to have a significant influence on bone mineral density (BMD), bone health also affects people’s social support networks. For example, older women with osteoporosis may have difficulty making and maintaining a large social relationship. Therefore, variables such as impairment in the activities of daily living (ADL) or instrumental activities of daily living (IADL) could be controlled for to minimize reverse causation. ADL was composed of 6 items, such as bathing, dressing, feeding, rising or lying down, walking, and toileting, and IADL was composed of 8 items, such as shopping, using a phone, using public transportation, doing light house work, doing laundry, preparing meals, taking medicine, and managing money. In this study, we controlled for the IADL in regression models, although the same results were observed when ADL was controlled for (results are not shown). In addition, we also examined the model in which those with impaired IADL were excluded as another way to minimize the reverse causation.

### Data analysis

Statistical analyses were performed as follows. First, bivariate analyses were performed to examine the association between the incidence of osteoporosis and various covariates such as age, BMI, drinking, smoking, household income, education level, IADL, and living area, in addition to social network characteristics such as marital status, social support network size, and average intimacy level. To examine statistical difference between those who have osteoporosis and those who do not have osteoporosis, t-tests were conducted for continuous variables, and chi-squared tests were performed for categorical variables. Next, three logistic regression models were used to predict the presence of osteoporosis. The first model predicted the presence of osteoporosis by the social support network size, adjusted for several covariates that were confirmed to be significant in previous studies. In this model, to examine the nonlinear influence of social support network size, a squared term of the social support network size was also included. In the second model, to minimize the reverse causation of social support network size on the incidence of osteoporosis, IADL was additionally added. The last model tested the contingent association between the social support network size and osteoporosis. Thus, the interaction between the social support network size and average intimacy level was examined to reveal how the association between the social support network size and osteoporosis varies based on the average intimacy level. Throughout the paper, statistical significance was tested using the criteria of p-value < 0.05.

This study was approved by the Institutional Review Board of Severance Hospital at Yonsei University College of Medicine (approval number: 4-2012-0172; approval date: May 3 2012).

## Results

Among the 1,955 female participants, after excluding those with missing values for study variables, 1,926 subjects remained. Additionally, because this study aimed to examine the contingent effect of the social support network size by the average intimacy level, 80 women who had no social support network members were excluded from the study, because the intimacy level could not be defined for respondents without any social ties. The final sample therefore included 1,846 female participants.

The second row of the Table 1 presented the descriptive statistics of the study participants. About 60% of the subjects were currently married. Concerning the social support network size, 23% of the subjects have 1, 24% of them have 2, 24% of them have 3, 15% of them have 4, 8% of them have 5 and 4% of them have 6 social support network members. Average age was about 71, and average BMI was about 24. About 75% of the subjects did not drink, and about 97% of them did not smoke. About 60% of the subjects do exercise more than once a week. About half of the subjects have annual income less than 10,000 dollar, and about 25% of them have annual income 10,000 to 20,000 dollar and the rest 25% have annual income more than 20,000 dollar. About 28% of the subjects did not graduate elementary school, about 36% of them finished elementary school, and about 36% of them finished middle school or higher education. Most of the subjects did not have impairment in instrumental activities of daily living. About 83% of the subjects lived in urban area and 17% of them lived in rural area. About 25% of the subjects had fracture history.

**Table 1:** General characteristics of study subjects.

Variables	All Participants n = 1,846	No Osteoporosis (T-Score > -2.5) n = 1,018	Osteoporosis (T-Score ≤ -2.5) n = 828	p-value
**Marital status**				
**Currently not married**	722 (39.11%)	363 (50.28%)	359 (49.72%)	0.001
**Married**	1,124 (60.89%)	655 (58.27%)	469 (41.73%)	
**Network size**				
**1**	441 (23.89%)	210 (47.62%)	231 (52.38%)	
**2**	447 (24.21%)	238 (53.24%)	209 (46.76%)	
**3**	451 (24.43%)	258 (57.21%)	193 (42.79%)	< 0.001
**4**	275 (14.90%)	179 (65.09%)	96 (34.91%)	
**5**	155 (8.40%)	93 (60.00%)	62 (40.00%)	
**6**	77 (4.17%)	40 (51.95%)	37 (48.05%)	
**Average intimacy level**	2.19 ± 0.73	2.22 ± 0.71	2.15 ± 0.75	0.04
**Age (years)**	71.29 ± 4.47	70.36 ± 4.03	72.42 ± 4.73	< 0.001
**Body mass index (kg/*m***^**2**^**)**	24.48 ± 3.07	25.15 ± 3.04	23.64 ± 2.90	< 0.001
**Drinking alcohol**				
**No drinking**	1,394 (75.51%)	754 (54.09%)	640 (45.91%)	
**Less than once a week**	350 (18.96%)	204 (58.29%)	146 (41.71%)	0.28
**More than once a week**	102 (5.53%)	60 (58.82%)	42 (41.18%)	
**Exercise**				
**No exercise**	712 (38.57%)	340 (47.75%)	372 (52.25%)	
**Once to four times a week**	538 (29.14%)	315 (58.55%)	223 (41.45%)	< 0.001
**More than five times a week**	596 (32.39%)	363 (60.91%)	233 (39.09%)	
**Smoking status**				
**Non-smoker**	1,786 (96.75%)	992 (55.54%)	794 (44.46%)	
**Ex-smoker**	33 (1.79%)	19 (57.58%)	14 (42.42%)	0.01
**Current smoker**	27 (1.46%)	7 (25.93%)	20 (74.07%)	
**Household income**				
**Less than 10,000 dollars**	917 (49.67%)	429 (46.78%)	488 (53.22%)	
**10,000 to 20,000 dollars**	474 (25.68%)	292 (61.60%)	182 (38.40%)	< 0.001
**More than 20,000 dollars**	455 (24.65%)	297 (65.27%)	158 (34.73%)	
**Educational level**				
**No schooling**	523 (28.33%)	238 (45.51%)	285 (54.49%)	
**Elementary school**	657 (35.59%)	355 (54.03%)	302 (45.97%)	< 0.001
**Middle school or higher**	666 (36.08%)	425 (63.81%)	241 (36.19%)	
**Instrumental activities of daily living**				
**Not impaired**	1,750 (94.80%)	972 (55.54%)	778 (44.46%)	0.01
**Impaired**	96 (5.20%)	46 (47.92%)	50 (52.08%)	
**Living area**				
**Urban**	1,538 (83.32%)	886 (57.61%)	652 (42.39%)	< 0.001
**Rural**	308 (16.68%)	132 (42.86%)	176 (57.14%)	
Fracture history*				
**Yes**	466 (25.24%)	215 (21.12%)	251 (30.31%)	<0.001
**No**	1,380 (74.76%)	803 (78.88%)	577 (69.69%)	

The second row shows the descriptive statistics of the entire participants (n = 1,846). The 1,846 elderly women were divided into two groups, those who had osteoporosis (n = 828) and those who did not (n = 1,018), using a T-score of -2.5 as a cutoff point. Either the t-test or chi-squared test was performed to examine whether these two groups had different characteristics. P-values are shown for t-tests or chi-squared tests. For continuous variables, means and standard deviations are reported; for categorical variables, frequencies and percentages are reported. The exchange rate is approximately 1000 Korean won for 1 US dollar.

* Distribution of fracture experience was presented by column to show the difference between normal and osteoporosis group, while distributions of other variables were presented by row.

Among the 1,846 subjects, 828 (44.9%) were diagnosed with osteoporosis. In the bivariate analyses presented in Table 1, currently married older women were less likely to be diagnosed with osteoporosis compared to the currently not married older women (p = 0.001). The distribution of osteoporosis patients was significantly different according to the social support network size (p < 0.001). The odds of having osteoporosis decreased with the presence of more social support network members until four network members. Beyond this threshold, the odds of osteoporosis increased with larger social support network size. As a result, we observed a U-shape association between the size of social support networks and the odds of having osteoporosis. Osteoporosis was significantly more common among those with less intimate social networks, older age, and higher BMI. Exercise and smoking were significantly associated with osteoporosis, while drinking was not. The presence of a lower household income, lower educational level, IADL impairment, and rural residency were associated with osteoporosis. Finally, more elderly women have experienced fracture among the osteoporosis group, comparing to the normal group.

Table 2 shows three logistic regression models predicting the presence of osteoporosis. As shown in the first model of Table 2, the coefficients of both social support network size (p = 0.007) and squared social support network size (p = 0.01) were statistically significant. These results indicate that the relationship between the social support network size and osteoporosis was curvilinear and not merely linear, which is consistent with the results in Table 1. To minimize the risk of reverse causality, IADL impairment was additionally controlled for in the second model. Both network size (p = 0.007) and squared network size (p = 0.01) remained statistically significant after IADL was controlled for in the second model. By dividing the coefficient of the social network size (-0.4049) by double of the coefficient of squared social network size (0.0581) in the model and changing negative signs to positive, the threshold over which the social network effect reverses was estimated to be around 3.5. This shows that while moderately large social network size under four could be beneficial to the management of bone health, increasing members beyond four could offset this benefit. This result remained the same when those with impaired IADL were excluded from the model. Both social support network size (coefficient = -0.4082, p = 0.008) and squared social network size (coefficient = 0.0600, p = 0.01) were statistically significant (data not shown in the Table 2). This confirmed that our hypothesis was supported.

**Table 2:** The multiple logistic regression model presenting the curvilinear association between social support network size and osteoporosis (Defined by BMD ≥ 2.5 standard deviation from the mean for young adults).

	Model 1		Model 2		Model 3	
	Odds Ratio (95% Confidence Interval)	P—value	Odds Ratio (95% Confidence Interval)	P—Value	Odds Ratio (95% Confidence Interval)	P—value
**Marital status**						
**Currently not married (ref)**	1		1		1	
**Married**	1.0622 (0.846–1.333)	0.60	1.0621 (0.846–1.333)	0.60	0.9862 (0.780–1.247)	0.91
**Network size**	0.6671 (0.497–0.895)	0.007	0.6670 (0.497–0.895)	0.007	1.3405 (1.054–1.704)	0.02
**Network size squared**	1.0598 (1.012–1.109)	0.01	1.0598 (1.012–1.109)	0.01		
**Average intimacy level**					1.1128 (0.849–1.459)	0.44
**Interaction between network size and intimacy level**					0.8675 (0.783–0.962)	0.007
**Instrumental activities of daily living**						
**Not impaired (ref)**			1		1	
**Impaired**			1.0179 (0.643–1.612)	0.94	1.0148 (0.641–1.607)	0.95
**Age (year)**	1.0941 (1.067–1.122)	<0.001	1.0940 (1.067–1.122)	<0.001	1.0932 (1.066–1.121)	<0.001
**Body mass index (kg/*m***^**2**^**)**	0.8266 (0.798–0.857)	<0.001	0.8266 (0.798–0.857)	<0.001	0.8249 (0.796–0.855)	<0.001
**Drinking alcohol**						
**No drinking (ref)**	1		1		1	
**Less than once a week**	0.8901 (0.687–1.154)	0.38	0.8902 (0.687–1.154)	0.38	0.8619 (0.664–1.118)	0.26
**More than once a week**	0.9886 (0.637–1.535)	0.96	0.9888 (0.637–1.536)	0.96	0.9679 (0.622–1.507)	0.89
**Exercise**						
**No exercise (ref)**	1		1		1	
**Once to four times a week**	0.8931 (0.693–1.150)	0.38	0.8933 (0.694–1.151)	0.38	0.9277 (0.719–1.197)	0.56
**More than five times a week**	0.7698 (0.601–0.985)	0.04	0.7701 (0.601–0.986)	0.04	0.7863 (0.614–1.007)	0.06
**Smoking status**						
**Non-smoker (ref)**	1		1		1	
**Ex-smoker**	0.7255 (0.334–1.574)	0.42	0.7249 (0.334–1.574)	0.42	0.7038 (0.324–1.530)	0.36
**Current smoker**	2.5741 (0.977–6.784)	0.06	2.5715 (0.975–6.779)	0.06	2.6873 (1.030–7.011)	0.43
**Household income**						
**Less than 10,000 dollars (ref)**	1		1		1	
**10,000 to 20,000 dollars**	0.7041 (0.544–0.911)	0.008	0.7038 (0.544–0.911)	0.008	0.7035 (0.543–0.911)	0.008
**More than 20,000 dollars**	0.6679 (0.505–0.884)	0.005	0.6677 (0.504–0.884)	0.005	0.665 (0.502–0.881)	0.004
**Education level**						
**No school (ref)**	1		1		1	
**Elementary school**	0.9866 (0.761–1.280)	0.92	0.9871 (0.761–1.281)	0.92	0.9631 (0.742–1.250)	0.78
**Middle school or higher**	0.714 (0.536–0.951)	0.02	0.7145 (0.536–0.952)	0.02	0.6912 (0.518–0.922)	0.01
**Living area**						
**Urban (ref)**	1		1		1	
**Rural**	1.2893 (0.962–1.728)	0.09	1.2898 (0.962–1.729)	0.09	1.3609 (1.013–1.829)	0.04

In total, 1,846 subjects were included in the model. Odds ratios, 95% confidence intervals, and p-values are reported for each variable.

**Fig 1:**
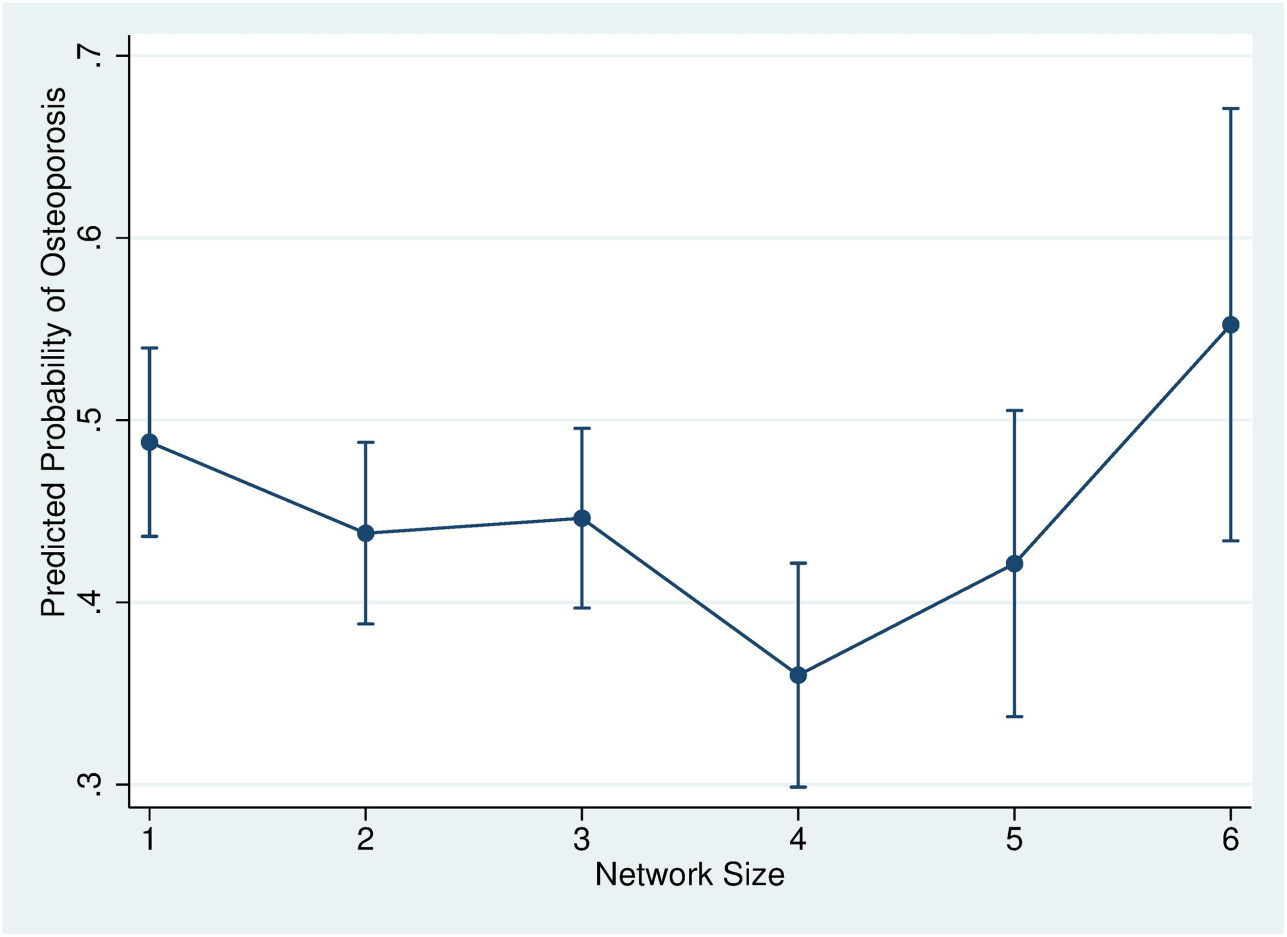
Probability of osteoporosis predicted by social support network size. The predicted probability of having osteoporosis was calculated for each social support network size. The estimated predicted probability was based on the logistic regression which was adjusted for other covariates such as marital status, age, BMI, drinking, smoking, exercise, household income, educational level, living area, and instrumental activities of daily living. Vertical lines indicate 95% confidence intervals.

Fig 1 graphically showed the predicted probability of osteoporosis for each social support network size when covariates in model 2 were adjusted as mean. When social support network size was 1, the predicted probability of osteoporosis was 47.8%. The predicted probability decreased into 43.8% when size was 2, 44.6% when size was 3, and 36.0% when size was 4. However, the predicted probability of osteoporosis increased into 42.1% when size was 5. When size was 6, the predicted probability was 55.2%, which was even higher than the predicted probability for size 1.

**Fig 2:**
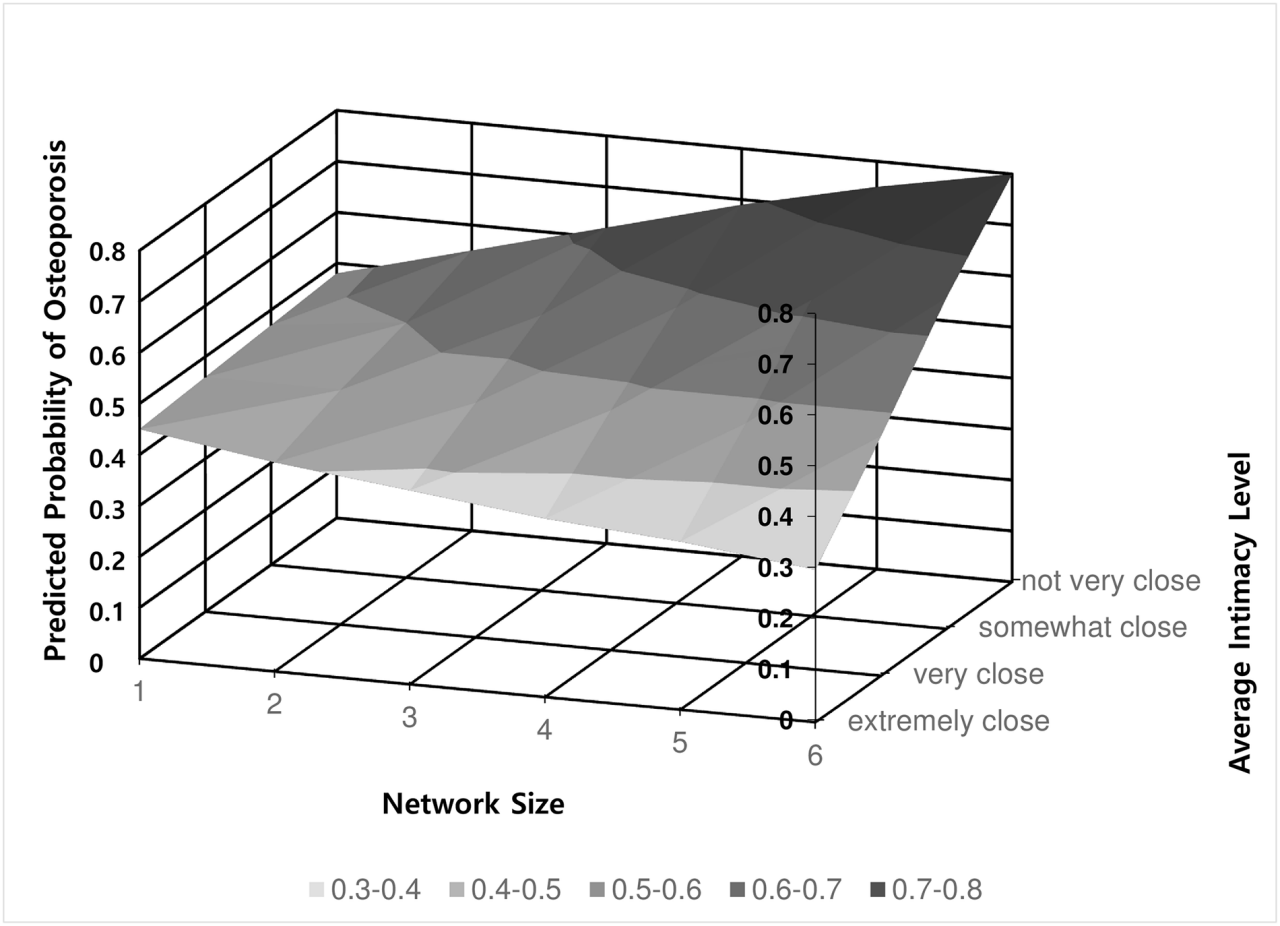
Probability of osteoporosis predicted by the social support network size and average intimacy level. The predicted probability of having osteoporosis was calculated for each social support network size and average intimacy level. The estimated predicted probability was based on the logistic regression which was adjusted for other covariates such as marital status, age, BMI, drinking, smoking, exercise, household income, educational level, living area, and instrumental activities of daily living.

To test the second hypothesis that the association between the social support network size and osteoporosis incidence is contingent on the average intimacy level of social networks, the interaction term between the social support network size and average intimacy level was added to the regression model. Results of the third model presented in Table 2 showed a statistically significant interaction between the social support network size and average intimacy level (p = 0.007). Although not presented in Table 2, the model excluding individuals with impaired IADL showed the same results. This contingency is displayed in Fig 2 for easier interpretation. Fig 2 shows the predicted probability of having osteoporosis depending on the size and average intimacy level of social networks among older Korean women, after controlling for all other variables in model 3 as mean values. This depiction shows that not all large social networks are beneficial: large networks are valuable only when the networks are intimate. When the average intimacy level was “extremely close”, larger networks lowered the risk of osteoporosis from 45% to 30%. However, if average intimacy level was “not very close”, larger networks increased the risk from 48% to 80%. Therefore, the lowest risk of osteoporosis was found among the group with the largest network size and the highest intimacy level, while the highest risk was observed for those with the largest network size and the lowest intimacy level. The result strongly supported our second hypothesis.

## Discussion

In general, the social support network is associated with lower risk of osteoporosis. Other individuals in older women’s social support network can provide them with various forms of social support. For example, they can provide instrumental help in older women’s daily lives, financial aids or useful information. They can also provide emotional support, so that older women can better manage their health. All these types of social support can decrease or buffer depression and stress, which can increase osteoclast activity and decrease osteoblast activity [34,35] Additionally, inflammatory markers that increase in a depressed state are considered to be associated with low bone mass [36,37]. Social support networks also have been known to encourage behaviors such as smoking cessation [38], leisure-time physical activity [39], and dietary habits [40], which have been known to be protective factors. People with large social support networks are more likely to consume calcium and vitamin D, and to engage in outside activities with more frequent sun exposure. Considering the common problem of lethargy in older adults, social participation in general provides considerable benefits.

However, the beneficial effect of the social support network is valid only when the social network is of a manageable size. Increasing one’s number of friends over a certain threshold can lead to mental exhaustion, perhaps because too much time and effort is required to maintain these relationships [12,41]. When people are embedded within large but less-intimate social support networks, their social ties may be burdensome rather than a helpful. Relationships may therefore become stressful or irritating [13–15] and can cause envy, invasion of privacy, or interpersonal conflicts [16–19].

To our knowledge, this is the first study to discover the negative association between maintaining large social relationships and the incidence of osteoporosis. Previous studies have mainly focused on how maintaining specific social relationships, particularly being currently married or living with spouses, can help prevent osteoporosis [7–10,42]. This study expanded the understanding of the association between social support and osteoporosis by examining social networks beyond spousal relationships.

These findings have important implications for the prevention of osteoporosis. Although maintaining good spousal relationships is clearly beneficial, older women who cannot maintain their marital relationship can still lower their odds of having osteoporosis by forming and keeping a large and intimate group of supportive friends. The estimated probability of having osteoporosis after controlling for other variables in Table 2 for non-married older women with large and close support networks was about 38% while married older women with small support networks is about 42%. This suggests that the disadvantage of not having a spouse may be overcome by maintaining a large and close supportive network.

Our result could help to identify a hidden risk group that was largely ignored in the previous health care programs such as osteoporosis liaison service [43–45]. Older adults who successfully maintain large size of social relationship were believed to enjoy many health benefits but our result clearly showed that too many not-so-intimate social relationships could increase the probability of having osteoporosis. Many preventive programs could identify this new type of risk group by utilizing our result.

Despite its contributions, this study had three major limitations. First, as it was based on cross-sectional data, the ability to make a causal inference is limited because bone density was measured at one point in time, rather than the change in bone density over time. The statistical association between osteoporosis and the social support network size may therefore be explained by other factors. For instance, it might be attributed to the fact that poor bone health causes small or less intimate social support networks. It can also be explained by compounding effects that cause both osteoporosis and small or less intimate social support networks. Although we attempted to minimize reverse causation by controlling for IADL in the regression model, we still believe that our estimated network effects may have been overestimated. Furthermore, unobserved variables that might have affected both bone density and social support networks could not be controlled for in the analysis. Assuming fixed effects using panel data would have put us in a better position to minimize the risk of reverse causation between the size of social networks and the incidence of osteoporosis. Because KURE was designed to track original respondents and become a prospective dataset, this limitation is expected to be overcome in the near future.

The second limitation is that this study could not empirically show the mechanisms by which social support networks and osteoporosis are associated among older Korean women. Although this study discovered that social support networks have a contingent association with osteoporosis, the behavioral, psychological or physiological pathways linking the two are still missing. Further studies are needed to clarify the specific mechanisms involved.

The final limitation was the lack of generalizability of findings. Several characteristics, such as BMI, smoking and alcohol related behaviors, were similar with those of general Korean older women [42], However, the elderly participants were invited to the hospital to participate in the study, only those who were healthy enough to come to the hospital were included. As a result, disabled patients with an extremely low BMD were systematically excluded from our data, causing a limited patient-level data issue. In fact, the prevalence of osteoporosis in the study was approximately 8% point lower than the national prevalence in Korean women aged over 60 years (52.6%) [42]. Moreover, the recruitment was conducted in limited specific region of Korea, making it difficult to generalize to all older Korean women. Further research is required using a nationally representative sample to test the generalizability of our findings.

To summarize, this study revealed that having a social support network has both positive and negative association with osteoporosis among older Korean women. Specifically, the relationship is contingent on the average intimacy level of relationships within the network. While older women with large networks and high average intimacy levels receive the most benefit from their social networks, those with large but less intimate social networks are at the highest risk of having osteoporosis.
